# Predictors of need for help with weight loss among overweight and obese men and women in the Netherlands: a cross-sectional study

**DOI:** 10.1186/s12913-017-2759-1

**Published:** 2017-12-12

**Authors:** S. N. W. Bunt, S. Y. M. Mérelle, I. H. M. Steenhuis, W. Kroeze

**Affiliations:** 1 VU University Amsterdam, de Boelelaan 1085, Amsterdam, The Netherlands; 2 Municipal Health Service Kennemerland, Zijlweg 200, Haarlem, The Netherlands

**Keywords:** Perceived need for help, Help-seeking, Weight loss, Overweight and obesity, Predisposing characteristics, Enabling resources

## Abstract

**Background:**

Need for help is perceived as an important first step towards weight related health-care use among overweight and obese individuals and several studies have reported gender as an important predisposing characteristic of need for help. Therefore, the goal of the current study is to gain insight into factors that might determine need for help for weight loss in men and women.

**Methods:**

In the current study, data from the Dutch cross-sectional survey Health Monitor 2012 was used. Overweight and obese men (*N* = 2218) and women (*N* = 2002) aged 19–64 years were selected for the current study. Potential predictors of need for help were age, ethnicity, marital status, educational level, perceived health, weight status, comorbidities, physical activity level, and income. Multiple logistic regression analyses were conducted separately among men and women to establish prediction models of need for help for weight loss.

**Results:**

The mean age of the adult women in this study population was 47.7 years and 68% was medium educated, whereas the mean age of men was 49.0 years and 63.0% was medium educated*.* Of the respondents, 24.9% indicated they either felt a need for help for weight loss, 6.4% already received help and 68.7% felt no need for help. Women were more likely to indicate a need for help than men (OR = 2.17). Among both genders, need for help was significantly predicted by obesity (OR_men_ = 3.80, OR_women_ = 2.20) and “poor” perceived health (ORmen = 2.14, OR_women_ = 1.94). Besides, “unmarried” (OR_men_ = 1.57) and suffering from comorbidities (OR_men_ = 1.26) predicted need for help among men. Whereas among women, need for help was predicted by younger age (i.e. 19–34 years (OR_women_ = 2.07) and 35–49 years (OR_women_ = 1.35)).

**Conclusion:**

The current study revealed specific predictors of need for help for weight loss for men and women. Among men, the strongest predictors were obesity and poor perceived health, whereas among women need for help was most strongly predicted by obesity and young age. Insight into these specific predictors enables health professionals to reach overweight individuals with a need for help for weight loss by connecting their need to available support.

## Background

Worldwide, a great public health concern is the high prevalence of overweight and obesity in adults [1]. Prevalence of overweight adults increased with almost 10 % between 1980 and 2013. More specifically, in 2012, nearly half of the Dutch adult population was overweight or obese (Body Mass Index ≥25 kg/m^2^) [2]. Moreover, 53.0% of Dutch males were overweight [3] as opposed to 43.7% of Dutch females [4] while the prevalence of obesity was a little higher in Dutch women (13.9%) compared with men (11.3%) [5, 6]. Overweight and obesity are associated with several chronic diseases, such as cardiovascular diseases, specific types of cancer, and type II diabetes [7, 8].

In recent years, researchers in the field of science of overweight and obesity have extensively studied causes for these problems. They have been established to be multi-factorial problems caused by energy-balance related behaviours (i.e. dietary behaviours and physical activity) and multi-type (i.e. physical, political, economic, sociocultural) environmental factors which individuals come across at either micro- or macro-level (e.g. neighbourhoods and health systems, respectively) [9]. This multi-faceted character of overweight often prohibits overweight individuals to make positive behaviour changes and tackle their health problems, despite having the necessary knowledge to positively change their energy-balance related behaviours [10]. Therefore, appropriate professional or social support from different sources is proposed to resolve some of these barriers to healthy behaviour changes [11].

Despite the fact that the problems of overweight and obesity are difficult to address without help and supervised weight loss might be more effective [12], research indicates that the majority of overweight individuals who were ready to lose weight wanted to do this on their own [13, 14]. Previous research indicated overweight individuals perceived many barriers for the help from a doctor or professional and the least barriers for treatment on their own [15]. This indicates that the need for help for weight loss among overweight individuals is low. Furthermore, only 2% of the Dutch adult population made use of weight-related care in 2010 [16]. The limited use of weight-related care in combination with a higher preference for “treatment on own” indicates that need for help might be an important indicator for weight-related health-care use.

Based on the Model of Health Services by Andersen [17], and on previous research on help-seeking behaviour in another chronic condition, this study assumes health care use is a product of, among others, individual characteristics. The current study focuses on these individual characteristics as a first step in understanding need for support for weight loss. Specifically, the individual perceived need for help for weight loss, as opposed to the clinical or objective need for weight loss. The following determinants are hypothesized to play a role in perceived need for weight loss: 1) predisposing characteristics (e.g. demographics, socio-economic status), 2) health- and weight-related factors (perceived health status, comorbidities, Body Mass Index (BMI), physical activity), and 3) enabling resources (e.g. financial factors).

Several studies have reported gender differences as an important predisposing characteristic in health care use [13, 18]. However, to date no studies have discussed factors predicting need for help regarding weight-related care, while evidence from other disciplines such as treatment for substance use disorders and mental health problems indicate the relevance of gender differences in need for help. [19–21]. The current study builds on insights into weight-related care use provided by Tol et al. [13]; it adds to it by examining need for help among a larger and more representative adult population. Furthermore, it explores need for help separately among men and women. The aim of the current study therefore is to identify which predisposing characteristics and enabling resources predict need for help for weight loss separately among adult men and women with overweight and obesity in the Netherlands.

## Methods

### Study design and population

This study concerned a secondary analysis from the cross-sectional survey Health Monitor 2012 carried out by the Municipal Health Service Kennemerland in the Netherlands. This survey was conducted to provide insights into the physical and mental health and lifestyle risks of adults and senior citizens of Kennemerland. Participants were randomly sampled from municipality registries [22]. Based on a sample calculation which took into account the size of the total target population as well as the expected response rate, 23,430 adults in the age of 19 to 65 years old were approached [22]. Individuals were excluded if they lived in nursing homes, mental institutions or institutions for the mentally disabled, or if they were imprisoned or were staying at asylums. In total, 9112 invited adults participated (response rate 39%). For each of the following subgroups the minimum response rate of 20% was achieved: men 19–34 years, men 35–49 years, men 50–64 years, women 19–34 years, women 35–49 years, women 50–64 years, Native Dutch, Western and non-Western immigrants [23]. In the current study, only the overweight and obese adult respondents were included. Of the total adult respondents 4220 respondents were overweight or obese (BMI ≥25).

### Procedure

Data were collected from October till December 2012. Selected individuals were invited by postal invitation to participate in an online survey. Those who did not reply within two weeks received their log-on codes for the online survey as well as a paper-based version of the survey. A second reminder was sent again after two weeks and included log-on codes and the possibility to request a paper-based survey. Privacy of the respondents was warranted by separating the data used to communicate with respondents from the research dataset. Secondly, communication of study data output to others occurred anonymously. Data from the survey were enriched by linking it to other datasets of Statistics Netherlands regarding income, ethnicity, year of birth and gender [23]. This linking of data was permitted because participants were made aware of the linking in the invitational letter they received. Under Dutch law, ethical approval was not necessary for this secondary analysis of anonymous data (23).

### Measurements

For assessing the need for help for weight loss and other health problems, a questionnaire was developed by GGD Amsterdam [24]. This questionnaire was pre-tested among 18 adults with various educational levels. Based on their evaluations, improvements were made regarding clarity and comprehensibility of the questions [25]. Need for help for weight loss was defined as contact with a health care worker or doctor, following a program/course, going to a general practitioner consultation or having conversations with fellow sufferers. Answering options included a) “yes, certainly”, b) “yes, maybe”, c) “no, since I already receive help”, and d) “no need for help”. These were dichotomized into “need for help” (a, b and c) and “no need for help” (d) to guarantee sufficient subgroup sizes for analyses. Further, information on gender was obtained from a question in the survey and from data of Statistics Netherlands. When participants did not report their gender in the survey, it was obtained from data of Statistics Netherlands.

In the current study, age and ethnicity were obtained by linking data of the Health Monitor 2012 to datasets of Statistics Netherlands. Age was categorized into 19–34 years, 35–49 years and 50–64 years. Ethnicity was categorized into three categories: Native Dutch, Western and non-Western immigrant. This classification is a standard way of classifying ethnic backgrounds in research of Statistics Netherlands [26]. Further, marital status was asked and answering options were “married”, “cohabiting”, “divorced”, “widowed” and “unmarried”. “Married” and “cohabiting” were recoded in the category “married/cohabiting”. Participants were further asked about their highest completed education. Answering options ranged from “no education”, through different levels of Dutch education and training programs, to “university degree”. These educations were divided into low (primary, lower vocational), medium (secondary, secondary vocational), and high educational level (≥bachelor’s degree).

Perceived health was asked in a single question, which has been proven to provide predictive validity of health outcomes [27]. The answer was given on a 5-point scale ranging from “very good” to “very bad”. This variable was dichotomized into good (i.e. “very good” and “good”) and poor health (i.e. “fair”, “bad”, and “very bad”). Weight and height were measured by self-reporting of respondents. Subsequently, mean Body Mass Index (BMI) (kg/m^2^) was calculated and was dichotomized into “overweight” (25 ≤ BMI < 30) and “obese” (BMI ≥ 30). Further, parts of the validated and reliable SQUASH questionnaire were used to measure physical activity level [28] and physical activity levels were categorized based on the Dutch norm for sufficient physical activity (i.e. ≥5 days/week 30 min moderately intense physical activity). Participants were categorized into “non-active” (never meeting the norm), “semi-active” (meeting the norm 1–4 days/week) and “norm active” (meeting the norm at least 5 days/week).

The following comorbidities were included in the present study: cardiovascular diseases (CVD), diabetes, all cancers, asthma or COPD, gastrointestinal diseases, osteoarthritis and back conditions. Questions in the survey on chronic diseases were based on the standard questionnaire on chronic conditions developed by Statistics Netherlands [29]. CVD was measured in five questions on the following topics: heart attack, serious heart condition, stroke, high blood pressure, and narrowing of the blood vessels in the abdomen or legs. Participants were categorized in “no CVD” (answering “no” to all questions) and “having CVD” (answering “yes” to one or more questions). Osteoarthritis was asked in two questions related to osteoarthritis and chronic inflammation of the joints. Upon answering “yes” to either question, respondents were categorized as “having osteoarthritis”, if not they were categorized as “having no osteoarthritis”. For the comorbidities cancer, asthma and COPD, gastrointestinal diseases, diabetes, and back conditions, participants were asked if they had any of these conditions in the last 12 months (yes/no). Abovementioned chronic conditions were added together in one variable; “comorbidities” with a “yes” (≥1 chronic condition) and a “no” (no chronic condition) category.

Statistics Netherlands provided standardized data on income of respondents which was defined as the net yearly earnings of the participant’s household without any benefits and was corrected for differences in household size and composition [30]. Answering options were as follows: a) ≤ €15,200; b) €15,201–€19,400; c) €19,401–€24,200; d) €24,200–€31,000; and e) > €31,000. For the current study standardized household income was further categorized into low (≤€15,200–€19,400), middle (€19,401–€31,000), and high income (>€31,000) [30].

### Statistical analyses

First of all, patterns of missing data were analysed using SPSS version 22.0, which revealed a random pattern of missing data in 86.4% of the variables and in 16.5% of the cases. To handle this pattern of missing data, multiple imputation was used and ten imputations were created according to guidelines of the EMGO+ Institute [31]. The pooled summary measure of the ten imputations was used in the subsequent analyses. Secondly, descriptive analyses were conducted to examine the distribution of the different variables among the study population separately for men and women. Significant differences between men and women regarding these variables were analysed using either independent samples t-tests or logistic regression analyses, depending on distribution of the variable. *P*-values <0.05 were considered statistically significant. Third, a logistic regression analysis was used to analyse if women were more likely to indicate a need for help for weight loss than men. Finally, to establish predictors of need for help, multiple logistic regression analyses were conducted separately for men and women. In each model the selected factors were firstly included together in a multivariate model. Secondly, using a backwards selection procedure the final model was formed and included the strongest predictors of need for help for weight loss (*p* < 0.05) [32]. The effect sizes of the associations were classified as minimal (OR < 1.5), small (1.5 ≤ OR < 2), medium (2 ≤ OR < 3) and large (OR ≥ 3) [33, 34]. The two latter classifications were considered practically relevant. Lastly, the accuracy of the prediction model was classified using the traditional academic point system of the area under the curve (AUC) of the ROC-curve; excellent (0.9–1.0), good (0.8–0.9), fair (0.7–0.8), poor (0.6–0.7) and fail (0.5–0.6) [35].

## Results

### Study population

As shown in Table 1, the study population consisted of 4220 adults, of which 2002 women and 2218 men, with a mean age of 48.4 (SD = 11.3). Furthermore, the study population predominantly consisted of respondents with a native Dutch background (85.3%) and respondents who were married or cohabiting (79.1%). Regarding other variables, the average BMI of the respondents was 28.5 (SD = 3.3) and a quarter had obesity. Further, six out of ten respondents met the Dutch norm for sufficient physical activity. One fifth of the study population described their general health status as poor, and over half of the respondents suffered from one or more weight-related chronic conditions. Further, men and women differed significantly on all variables besides ethnicity and physical activity.

**Table 1:** Characteristics of the overweight and obese adult study population, of the “Gezondheidsmonitor 2012” (*n* = 4220)

Variables	Categories	Men (n = 2218)	Women (*n* = 2002)	Total (n = 4220)
Need for help for weight loss, n (%)	Yes (certainly, maybe)	421 (19.0)	631 (31,5)	1055 (24.9)
	I already receive help	100 (4.5)	170 (8.5)	270 (6.4)
	No need for help	1697 (76.5)	1201 (60.0)	2898 (68.7)
Predisposing characteristics				
Age, mean (SD)		49.0 (11.0)	47.7 (11.6)	48.4 (11.3)
Age category, n (%)	19–34 years	280 (12.6)	311 (15.5)	591 (14.0)
	35–49 years	743 (33.5)	674 (33.7)	1417 (33.6)
	50–64 years	1195 (53.9)	1017 (50.8)	2212 (52.4)
Ethnicity, n (%)	Native Dutch	1917 (86.4)	1681 (84.0)	3598 (85.3)
	Western	176 (7.9)	184 (9.2)	360 (8.5)
	Non-western	125 (5.6)	137 (6.8)	262 (6.2)
Marital status, n (%)	Married/ cohabiting	1789 (80.6)	1548 (77.3)	3337 (79.1)
	Unmarried	256 (11.6)	210 (10.5)	466 (11.0)
	Divorced	143 (6.4)	179 (8.9)	322 (7.6)
	Widowed	30 (1.4)	65 (3.3)	95 (2.3)
Educational level, n (%)	Low (primary, lower vocational)	91 (4.1)	108 (5.4)	199 (4.7)
	Medium (secondary, secondary vocational)	1398 (63.0)	1352 (67.5)	2750 (65.2)
	High (≥ bachelor’s degree)	729 (32.9)	542 (27.1)	1271 (30.1)
Health- and weight-related factors				
Perceived health, n (%)	Poor (fair, bad and very bad)	449 (20.2)	463 (23.1)	912 (21.6)
Body Mass Index, mean (SD)		28.1 (2.9)	28.9 (3.7)	28.5 (3.3)
Weight status, n (%)	Overweight (25 ≤ BMI < 30)	1778 (80.2)	1419 (70.9)	3197 (75.8)
	Obese (BMI ≥ 30)	440 (19.8)	583 (29.1)	1023 (24.2)
Comorbidities^b^, n (%)	≥ 1 weight-related chronic condition	1106 (49.9)	1105 (55.2)	2211 (52.4)
Physical activity level (PA)^a^, n (%)	Non-active (meeting PA norm 0 days/week)	134 (6.0)	119 (5.9)	253 (6.0)
	Semi-active (meeting PA norm 1–4 days/week)	722 (32.6)	615 (30.7)	1337 (31.7)
	Norm active (meeting PA norm ≥5 days/week)	1362 (61.4)	1268 (63.4)	2630 (62.3)
Enabling resources				
Income, n (%)	Low (≤€15,200–€19,400)	343 (15.5)	429 (21.4)	772 (18.3)
	Middle (€19,401–€31,000)	1041 (46.9)	958 (47.9)	1999 (47.4)
	High (>€31,000)	834 (37.6)	615 (30.7)	1449 (34.3)

^a^Physical activity level based on the Dutch norm for sufficient physical activity: ≥5 days/week 30 min moderately intense physical activity

^b^Comorbidities include: cardio-vascular diseases, diabetes, cancer, asthma/COPD, gastrointestinal diseases, osteoarthritis, and back conditions

Of the respondents, one third indicated they either felt a need for help for weight loss (24.9%) or they already received support (6.4%), together resulting in one “need for help” group (31.3%). Need for help was indicated by 40.0% of the female respondents of this study, as opposed to 23.5% of the male respondents. The pooled estimate of this analysis revealed a significant relationship between gender and need for help for weight loss: OR = 2.17 (95%-CI = 1.90–2.49), *p* < 0.001.

### Predictors of need for help among men

Table 2 shows the results of the logistic regression analysis used to predict need for help among men. Firstly, the multivariable model including all potential predictors revealed being unmarried, having a poor perceived health, being obese, and having comorbidities significantly predicted need for help. Further, an inverse relationship was found between need for help and medium educational level compared with high educational level. Finally, using a backwards elimination procedure, the final model demonstrated the following strongest predictors: unmarried (OR = 1.57, 95%-CI = 1.15–2.15), poor perceived health (OR = 2.14, 95%-CI = 1.65–2.78) and obesity (OR = 3.80, 95%-CI = 3.01–4.80). Although the variable “comorbidities” was included in the final model based on the backwards selection procedure, it did not significantly predict need for help for weight loss. Medium educational level did no longer predict need for help for weight loss in the final model. Further, the effect sizes of the final factors were classified. Practical relevance was only reached in the association between need for help for weight loss and the final predictors “poor health”, and “obese” (see Table 2, Effect size). Lastly, the accuracy of the model was fair based on an AUC of 0.7 (95%-CI = 0.66–0.72), *p* < 0.001 [35].

**Table 2:** Variables predicting need for help for weight loss among men – results of backwards selection procedure

Need for help for weight loss among men - yes or no (n = 2218, need for help among men, *n* = 521)					
	Multivariable				
	Basic model ^1^		Final model ^2^		Effect size ^3^
Variables^4^	OR	95% CI	OR	95% CI	
Predisposing characteristics					
Age 19–34 years	1.30	0.89–1.89			
Age 35–49 years	1.04	0.80–1.34			
Ethnicity (western)	1.00	0.67–1.50			
Ethnicity (non-western)	1.03	0.63–1.68			
Marital status (unmarried)	1.52 *	1.09–2.13	1.57 **	1.15–2.15	Small
Marital status (divorced)	1.30	0.85–2.00	1.27	0.83–1.93	
Marital status (widowed)	1.13	0.38–3.31	1.10	0.38–3.20	
Educational level (low)	0.90	0.51–1.57			
Educational level (medium)	0.74 *	0.58–0.94			
Health- and weight-related factors					
Perceived health (poor)	2.22 ***	1.70–2.91	2.14 ***	1.65–2.78	Medium
Weight status (obese)	3.98 ***	3.13–5.04	3.80 ***	3.01–4.80	Large
Comorbidities (≥1 weight-related chronic condition)	1.31 *	1.01–1.69	1.26	0.99–1.61	
Physical activity (non-active)	1.18	0.73–1.91			
Physical activity (semi-active)	0.82	0.64–1.06			
Enabling resource					
Income (low)	0.85	0.60–1.20			
Income (middle)	0.90	0.70–1.15			

* p < 0.05 ** *p* < 0.01 *** p < 0.001

^1^ Multivariable model

^2^ Final model including only the strongest predictors using a backwards selection procedure

^3^ Effect size was classified as minimal (OR <1.5), small (1.5 ≤ OR < 2), medium (2 ≤ OR < 3) or large (OR ≥ 3)

^4^ Reference group: age = 50–64 years; ethnicity = Native Dutch; marital status = married/cohabiting; education = high; perceived health = good; weight status = overweight; comorbidities = no comorbidities; physical activity = norm-active; income = high

### Predictors of need for help among women

Table 3 shows the prediction model of need for help for weight loss among women. The multivariable model revealed age groups 19–34 years and 35–49 years, poor perceived health, and being obese significantly predicted need for help. In the final model the following strongest predictors of need for help for weight loss were included: poor perceived health (OR = 1.94, 95%-CI = 1.55–2.43), age group 19–34 years (OR = 2.07, 95%-CI = 1.58–2.71), age group 35–49 years (OR = 1.35, 95%-CI = 1.09–1.66), and “obese” (OR = 2.20, 95%-CI = 1.79–2.70). Furthermore, effect sizes were classified and practical relevance was only reached among women in the association between need for help for weight loss and age category 19–34 years, and “obese” (see Table 3, Effect size). Finally, the accuracy of the final model for women was poor, based on an AUC of 0.64 (95%-CI = 0.62–0.67), *p* < 0.001 [35].

**Table 3:** Variables predicting need for help for weight loss among women – results of backwards selection procedure

Need for help for weight loss among women - yes or no (n = 2002, need for help among women, *n* = 801)					
	Multivariable				
	Model 1 ^1^		Model 2 ^2^		Effect size ^3^
Variables^4^	OR	95% CI	OR	95% CI	
Predisposing characteristics					
Age 19–34 years	2.11 ***	1.56–2.86	2.07 ***	1.58–2.71	Medium
Age 35–49 years	1.36 **	1.09–1.71	1.35 **	1.09–1.66	Minimal
Ethnicity (western)	1.03	0.74–1.43			
Ethnicity (non-western)	1.24	0.84–1.83			
Marital status (unmarried)	1.12	0.81–1.55			
Marital status (divorced)	1.06	0.75–1.50			
Marital status (widowed)	1.15	0.66–1.98			
Educational level (low)	0.77	0.47–1.26			
Educational level (medium)	0.89	0.71–1.11			
Health- and weight-related factors					
Perceived health (poor)	1.94 ***	1.51–2.49	1.94 ***	1.55–2.43	Small
Weight status (obese)	2.28 ***	1.85–2.82	2.20 ***	1.79–2.70	Medium
Comorbidities (≥1 weight-related chronic condition)	1.16	0.93–1.45			
Physical activity (non-active)	0.70	0.43–1.13			
Physical activity (semi-active)	1.09	0.88–1.35			
Enabling resource					
Income (low)	0.83	0.62–1.11			
Income (middle)	0.95	0.76–1.19			

* p < 0.05 ** p < 0.01 *** p < 0.001

^1^ Multivariable model

^2^ Final model including only the strongest predictors using a backwards selection procedure

^3^ Effect size was classified as minimal (OR <1.5), small (1.5 ≤ OR < 2), medium (2 ≤ OR < 3) or large (OR ≥ 3

^4^ Reference group: age = 50–64 years; ethnicity = Native Dutch; marital status = married/cohabiting; education = high; perceived health = good; weight status = overweight; comorbidities = no comorbidities; physical activity = norm-active; income = high

## Discussion

The current study aimed to identify predictors of need for help for weight loss among men and women. Results showed that men and women differed in their need for help for weight loss: women were more likely to indicate a need for help than men. Secondly, need for help for weight loss among men was predicted by a set of factors different from the set of factors predicting need for help among women. This indicates essential differences may exist between men and women regarding perceived need for help for weight loss.

Of the over four thousand overweight and obese respondents of the current study, three out of ten indicated a need for help for weight loss. This finding is promising in that it indicates that a substantial group of overweight individuals is in the “window of opportunity to change” as they feel a need for help for weight loss. The percentage of people with a need for help for weight loss in this study was slightly higher compared to previous studies [13, 15]. This may result from the use of a more general definition of need for help in the current study, thereby including more people with various forms of need for help for weight loss within one definition.

Regarding predictors of need for help for weight loss, results of the current study are largely in line with previous research. For men, the final model included the practically relevant predictors poor perceived health and obesity. Previous studies also found a higher desire and intention to lose weight in individuals with a higher weight [13, 15, 36]. Moreover, previous studies found associations between poorer self-perceived health and higher intention to use weight-related care and fewer weight misperceptions [13, 37]. Secondly, need for help was significantly predicted by being unmarried, which is similar to findings of previous studies regarding substance use disorders; unmarried individuals were significantly more likely to perceive a need for help or seek help than married individuals and were more likely to consult a health professional [19, 20]. This difference may be caused by relying on alternative sources of help among unmarried males, in comparison to married individuals who probably depend mostly on support of their spouses [19]. Finally, results of the current study showed among men suffering from one or more weight-related chronic conditions predicted need for help for weight loss. This could be due to the higher awareness of the health status and the health benefits of weight loss for those who suffer from chronic conditions and the medical advice given them to address their overweight [38, 39].

Regarding women a practically relevant predictor of need for help firstly was obesity, in line with previous studies [13, 15, 36]. A second practically relevant predictor was lower age (19–34 years). In addition, middle age (35–49 years) significantly predicted need for help. This is supported by results from previous research in which older women (>45 years) were less likely to report weight dissatisfaction and were less likely to report dieting than younger women [40]. This suggests older women are less interested in weight loss and would therefore be less likely to perceive a need for help for weight loss. Further, need for help was significantly predicted by poor perceived health, similar to results in men [13, 37]. The quality of the final model for women was poor, possibly because potential predictors of need for help could not be analysed in the current study, such as weight loss history [13], and psychological factors such as body image distress [41]. Furthermore, according to previous research, potential predictors of perceived need are health beliefs and social structural factors such as stigma and discrimination [17, 42–44]. These factors were not measured but might play a substantial role in perceived need for help specifically among women and should be included in future research.

Contrary to our expectations, ethnicity, educational level and income did not predict need for help for weight loss [13, 41, 45–47]. There are several explanations for these findings. Firstly, income might not have predicted need for help because Dutch health insurance at the time of the Health Monitor 2012 covered four hours of dietary treatment for obese individuals and individuals with overweight and comorbidities. Since a substantial part of the study population either consisted of obese individuals or individuals with one or more comorbidities, health insurance coverage might explain why lower income would not be a barrier to seeking help for weight loss among the study population, as also proposed in previous research [48]. Secondly, income, education and ethnicity might not predict need for help either because in fact no differences existed, or samples regarding these variables were too homogenous to detect differences (e.g. 86% native Dutch respondents without a need for help against 84% with a need for help). Further research is necessary to provide concluding evidence on need for help and income, education, and ethnicity.

Lastly, results of the current study showed women were significantly more likely to indicate a need for help for weight loss than men. Previous studies also showed that men generally made less use of weight-related health care compared with women [13, 18]. Possible explanations for these differences are derived from studies conducted on related topics, as this was beyond the scope of the current study. First of all, lower need for help among men could be due to the fact that men underestimate their weight [36, 49–51] and are less likely to recognize their weight as a health risk compared with women [52]. A second explanation could be that in general men put lower priority on health behaviour and report less health promotion behaviours than women [45, 53]. When weight management and health promotion is prioritized low, this could result in lower need for help. Lastly, a cause of gender differences in need for help might be found in the traditional gender-role: men might associate seeking help with weakness and might associate showing vulnerability with decreased masculinity [54, 55].

### Strengths and limitations

The results of the current study should be considered in light of several limitations. Firstly, because of the cross-sectional design of the current study no statements can be made about causality of the relationship between need for help and determinants of help-seeking behaviour. A second limitation is that no distinction could be made between specific types of help for weight loss, as the question related to need for help did not allow participants to specify their preferences. Still, general perceived need for help for weight loss is an important first step in help-seeking behaviour and thereby a first step in identifying individuals who are in the window of opportunity to change.

Despite some limitations, the strengths of the current study must not be underestimated. First of all, to the best of our knowledge, this study is the first to identify specific predictors of need for help for weight loss for overweight and obese men and women, thereby providing a piece of the puzzle of the complex construct of the battle against overweight and obesity. A second strength is the large-scale and more representative study population of over four thousand overweight and obese adults compared with previous research [13, 56]. Similar to national distribution of gender, about half of respondents studied were female and the others were male. Moreover, similar to the national prevalence of overweight of 48.3%, in the current study 46.3% of adults was overweight or obese. Therefore, generalizability and representativeness were accomplished. Thirdly, a strong aspect of the current study is the fact that it is theory based and used a for the most part validated questionnaire consisting of questions which are in line with national guidelines. This increased the extent to which statements could be made with more certainty about need for help for weight loss. And lastly, conclusions were based on both the significance of *p*-values and on effect sizes, as the latter is considered to be more meaningful for practice [33, 34].

### Practical implications

This study contributes to the field of research related to help-seeking behaviour by creating a clear view of the group of overweight and obese men and women who indicate a need for help with losing weight. As a result, the current study makes some important contributions to practice. Firstly, although individuals with overweight are often stereotyped as lacking in motivation to change [57], results of the current study challenge this, as 31.3% of overweight or obese individuals did indicate a need for help for weight loss. This implies a substantial group of overweight individuals is willing to lose weight and are thus in the “window of opportunity to change”. However, since only 6.4% indicated to already receive help, this might suggest that barriers exist in accessing help with weight loss. To reach individuals in need of help, their need should be connected to available sources of help for weight loss. Opportunities for this connection could be offered for example in primary care by the general practitioner (GP) [13]. Currently, guidelines in the Netherlands regarding weight-related care are demand-driven (i.e. demands regarding weight or weight-related health problems) [58] and research indicated GPs perceive several barriers for initiating a discussion with their patients regarding their weight [59]. However, results of the current study indicate it could be beneficial for GPs to not only discuss weight-related care with patients with explicit demands regarding weight loss, but also discuss this with overweight patients who possess specific predictors of need for help identified by the current study. For chances are that those individuals might still feel a need for help for weight loss even without explicitly mentioning it to their GP.

## Conclusion

To conclude, the current study has established that a substantial group of overweight individuals perceive a need for help for weight loss. Furthermore, results showed women were more likely to indicate a need for help than men. Moreover, need for help among men was predicted by obesity, poor perceived health, marital status (unmarried) and comorbidities, whereas need for help among women was predicted by obesity, poor perceived health and younger age (<50). Insight into these specific factors predicting need for help among men and women could enable health professionals to connect need for help for weight loss to available sources of help. Further research is necessary to provide insight into the specific sources of help for weight loss preferred by overweight and obese individuals. Besides, as opportunities for connecting need for help for available sources could be provided primary care, future research should establish the effectiveness of these efforts. For when these effectively reach those individuals with a need for help for weight loss, it would yield substantial health benefits and would consequently result in significant reductions in the health care costs related to unhealthy lifestyles.

## Abbreviations

BMI: Body Mass Index
GP: General Practitioner
